# Gait and balance in cervical dystonia and dystonic head tremor

**DOI:** 10.3389/dyst.2023.11231

**Published:** 2023-08-14

**Authors:** Aparna Wagle Shukla, Anjela Gurrala, Vinata Vedam-Mai

**Affiliations:** Department of Neurology, Fixel Institute for Neurological Diseases, University of Florida, Gainesville, FL, United States

**Keywords:** dystonia, dystonic tremor, gait, walkway, essential tremor

## Abstract

**Background:**

Previous studies have found gait and balance abnormalities in patients with cervical dystonia. However, the characteristics of gait and balance in cervical dystonia with head tremors have not been ascertained. A midline constant head tremor when walking would likely render gait and balance more difficult. The pathophysiology of dystonia has also been increasingly linked with cerebellar function abnormality, commonly implicated in gait and balance disorders.

**Methods:**

We examined the gait and balance characteristics of cervical dystonia presenting with head tremors. We used the timed up-and-go (TUG) walk test, 10 m walk test, Berg Balance Scale (BBS), and Gait and Freezing questionnaire. We then assessed the gait on an instrumented walkway system to capture spatiotemporal measures such as speed, cadence, step time, step length, stride width, swing%, stance%, single support%, double support%, and gait variability index (GVI). We also assessed whether the gait in dystonic tremor (DT) differed from essential tremor (ET) and orthostatic tremor (OT), as these tremor disorders share the cerebello-thalamo-cortical pathway as the common pathological pathway.

**Results:**

50 participants comprising DT (20 patients), ET (15 patients), and OT (15 patients) were enrolled. While the gait abnormalities were subclinical, 11/20 DT patients (55%) walked at a slower speed on the TUG, 11/20 (55%) had reduced scores on the BBS, 9/20 (45%) had increased step time, 4/20 (20%) had reduced step length, 4/20 (20%) had wider stride width, 9/20 (45%) spent greater time during double support and 8/20 (40%) patients had an abnormal GVI. Comparisons of DT with healthy control data revealed a slower gait velocity (*p* = 0.001) and a reduced step length (*p* = 0.001). Compared to DT, the ET group revealed a reduced cadence (*p* = 0.04) and the OT group revealed an increased TUG time (*p* = 0.03), reduced BBS scores (*p* = 0.02), reduced step length (*p* = 0.02), reduced cadence (*p* = 0.03), reduced GVI (*p* = 0.01), and increased double support phase (*p* = 0.045).

**Conclusion:**

DT is accompanied by multiple abnormalities affecting gait and balance, albeit subclinical and less pronounced than ET and OT, possibly related to more effective compensatory mechanisms. Nevertheless, these abnormalities indicate that rehabilitative measures warrant consideration when managing in clinical settings.

## Introduction

Gait and balance difficulties can be seen in many tremor disorders, such as essential tremor (ET), Parkinson’s disease tremor, and orthostatic tremor (OT) [1–4]. The pathophysiology of these tremor disorders is linked with abnormalities of cerebellar functions, which are critical for gait and balance [5, 6], There is mounting evidence that the cerebellum is a key pathophysiological substrate in dystonia [7–9], thus implying that gait and balance could potentially be compromised in this patient population. As such, previous studies have found subclinical and clinical gait abnormalities in some forms of dystonia such as cervical dystonia [10]. It has been reported that these patients walk at slower speeds than healthy controls [11], and that they report a lower level of fall self-efficacy and balance confidence [11, 12]. However, to our knowledge, there are no studies that have ascertained and characterized the gait abnormalities in cervical dystonia when there is a co-occurring tremor affecting the head, referred to as the dystonic tremor (DT). A midline body tremor, especially a constant head tremor when walking, would plausibly render gait, balance and equilibrium more difficult.

Thus, in this study, we sought to characterize the gait in cervical dystonia patients presenting with dystonic head tremor. We used standardized clinical assessment questionnaires and scales for assessment of gait and balance and an instrumented walkway system for capturing individual spatiotemporal gait measures and compared these measures with data collected from age matched healthy controls. We ascertained whether the clinical features in patients with DT, such as the age, gender, disease duration, cognition, botulinum doses or head tremor severity, were related to the gait measures. We also assessed whether the gait characteristics in DT differed from those seen in patients with ET and OT as the cerebello-thalamo-cortical pathway was common and implicated in the pathophysiology for these tremor disorders.

## Methods

We prospectively enrolled DT, ET and OT patients in an IRB approved study who consecutively presented to our Movement Disorders Center at the University of Florida between 2019 and 2020. Diagnosis of DT, ET and OT was confirmed with clinical criteria following recommendations of the Movement Disorders Society [13]. We only enrolled those patients who were able to perform gait tasks comfortably and could walk on an instrumented walkway system while off medications and at least 3 months past their last botulinum toxin injections. We excluded patients with substantial arthritis, spinal disease and deformities, substance abuse, neuropathy symptoms and visual difficulties.

### Study protocol

Upon obtaining an informed consent, participants underwent a detailed clinical history assessment, and a complete tremor pertaining physical examination by a movement disorders specialist at the Fixel Movement Disorders Center. For the gait and balance component, participants were assessed with the following scales, tests and questionnaires: (1) Berg Balance scale (BBS); a 14-item objective measure for assessment of static balance and risk of falls in adults. BBS is used to objectively determine the subject’s ability (or inability) to safely balance during a series of predetermined tasks. Each item on the 14-item list consists of a five-point ordinal scale ranging from 0 to 4, with 0 indicating the lowest level of function and 4 indicating the highest level of function. The scale does not include the assessment of gait. High scores (50 and above) indicate normal balance (2) Time Up and Go (TUG) test; a test that captures transfers, gait, and turning movements used for the assessment of mobility, balance, walking ability, and fall risk. The test involves standing and sitting from a chair as well as walking a 3-meter distance. These components of the test allow examination of gait, turns, sit to stand, and turn to sit transitions. Most healthy controls need 10 s or less to complete the TUG test (3) 10 m walk test; a performance measure employed to assess walking speed measured in meters per second over a short distance. A gait speed < 1.1 m per second (m/s) is accepted to fall in the normal range. It can be used as a measure of functional mobility and gait. (4) Gait and Freezing Questionnaire (GFQ); a 6-item survey used to assess gait and freezing. The scale has two items specifically for assessment of gait. Response to each item is a 5-point interval scale ranging from 0 for the absence of symptoms to 5 for the highest severity of symptoms. Higher scores indicate an increased severity of impairment (5) Montreal Cognitive Assessment (MoCA); a screening technique designed to detect mild cognitive dysfunction. An impaired cognition can be seen in tremor disorders [14] and that can impact gait and balance.

Participants were then instructed to walk on a Zeno^™^ Walkway mat (ProtoKinetics, Havertown, PA) [20-foot-long x 4-foot-wide pressure sensor]. They were asked to sit with both feet placed on the ground on a chair that was 42 cm high was placed at the end of the gait mat. In response to an auditory cue, participants stood up and walked twice on the mat. Participants walked on the gait mat back and forth without breaks unless symptoms of unsteadiness precluded completion of the task. Four passes were recorded, and for each walking trial, data was collected at a sampling rate of 120 Hz (4 bits) for assessment of spatiotemporal parameters. Data was captured using the electronic, pressure-sensing walkway and analyzed using the ProtoKinetics Movement Analysis Software (PKMAS). The following gait outcome measures were collected and analyzed: speed (cm/s), distance traveled over time; cadence (steps/min), total number of steps per time period taken during a given time; step duration (s), time between the first contact of one foot; step length (m or cm), distance between two consequent footprints (heel) and stride width, distance between the feet while walking is the perpendicular distance between the line connecting the two ipsilateral foot heel contacts (stride) with the contralateral heel contact between those events (cm). Normal gait consists of two phases: the swing phase (40% of the gait cycle; when the foot first touches the ground and ends when the same foot leaves the ground) and the stance phase (60% of the gait cycle; when the foot first leaves the ground and ends when the same foot touches the ground again). These phases are divided into sub-phases; single limb support % involving mid and terminal stance subphase and double limb support % involving initial contact, loading, and pre-swing subphase. Finally, the gait variability index (GVI), a measure to quantify the variability in spatiotemporal variables, was collected (a score ≥100 indicates values similar to healthy controls, whereas a lower score denotes increased gait variability).

Statistical analysis was performed using IBM SPSS Statistics 27 (Armonk, NY). Demographics, baseline clinical measures, and gait assessments were compared between DT vs. healthy controls, DT vs. ET and DT vs. OT using Mann-Whitney U tests or χ^2^ tests as appropriate. In the DT group, continuous clinical measures were correlated with gait measures using the Spearman correlation test and the categorical predictors were analyzed with the help of Mann Whitney U test. The threshold for significance was set at *p*-value <0.05 and the Holm-Bonferroni method was used to correct for type I error rates for multiple comparisons.

## Results

50 participants comprising of DT (20 patients), ET (15 patients), and OT (15 patients) were enrolled.

### Demographics and clinical features of the DT cohort

15 females and 5 males participated. Mean age for the participants was 66.8 ± 10.8 (standard deviation or SD) years. Mean disease duration was 17.1 ± 13.2 years. Clinical characteristics of the DT cohort are presented in Table 1. All participants had a diagnosis of cervical dystonia with head tremor and except four participants, none endorsed clinical gait difficulties. Video segments of gait recorded for 2 DT patients is presented in Supplementary Information. Three DT patients had dystonia symptoms affecting the arm, two patients had laryngeal involvement, two had jaw and one patient had dystonia involving the eyes along with the neck. The mean severity of head tremor (based on the item 4 of Fahn Tolosa Marin tremor rating scale used routinely in our clinic) was noted to be 1.8 ± 0.6. Six participants had arm tremor, two had voice tremor and one had jaw tremor in addition to their head tremor. All participants except three were receiving botulinum toxin injections with mean dosage 231.5 ± 124.2 units. Gait assessment was performed when the participants were at least 3 months past their botulinum toxin injections and oral medications had been held off for at least 12 h. Thirteen patients were receiving benzodiazepines and two patients were receiving anticholinergics for dystonia. Nine patients were receiving betablockers and four patients were receiving primidone for treatment of tremor.

In the GFQ questionnaire, 4/20 patients (20%) were found to have abnormally elevated scores indicating that these patients reported some difficulties with walking. In the assessment of TUG time, a cut-off value of 12 s that has been found to differentiate fallers from non-fallers among the community-dwelling elders was used [15, 16]. We found with this cut-off, 8/20 participants (40%) needed more than 12 s and 3/20 (15%) patients needed more than 13.5 s to complete the task. 11/20 patients obtained slightly lower scores on the BBS test and in the 10 m walk test, 8/20 (40%) patients were observed to walk slow when a cut off score of 1.1 m/sec was used [17].

### DT gait on the instrumented walkway system

**FIGURE 1:**
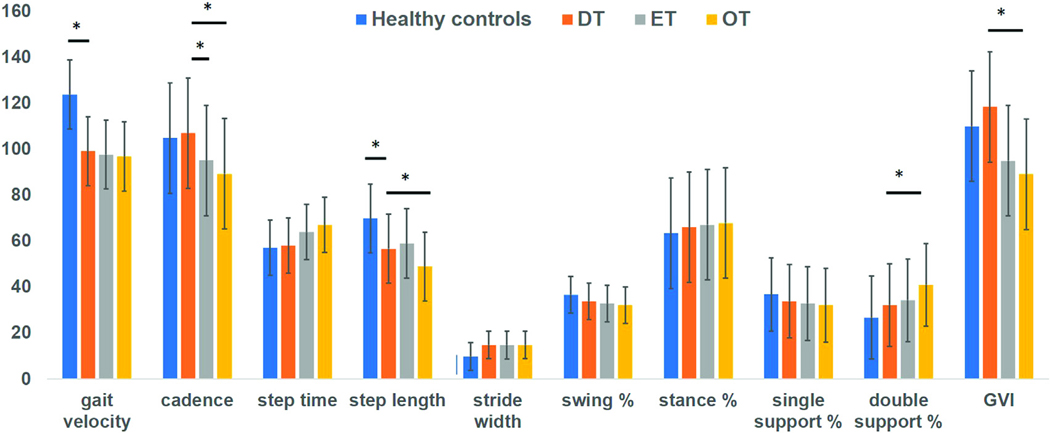
Bars represent mean values for gait data collected on instrumented walkway system. Absolute values of step time were multiplied by 100 to plot on the y axis (marked by asterisk). Blue bars represent gait data for healthy controls, orange represent gait data for DT cohort, grey represent gait data for ET cohort and yellow represent gait data for OT cohort. Error bars represent standard errors of mean. Stars placed between the bars in the data for gait velocity and step length illustrate significant differences between healthy control data and DT group.

Table 2 presents data for individual DT participants. The Supplementary Table presents data for age- and sex-matched healthy controls (*n* = 46). The minimum and maximum values for data collected from healthy controls within a specific age range is plotted in the Supplementary Table. When comparing against these values for healthy control data, 11/20 DT participants (55%) were identified to walk at a slower speed, 9/20 (45%) walked with increased step time; 4/20 (20%) walked with shorter step length; 4/20 (20%) had wider stride width, 6/20 (30%) participants had shorter time spent during the swing phase; 7/20 (35%) had reduced time spent during single support, 9/20 (45%) spent greater time during double support and 8/20 (40%) patients had an abnormal gait variability index. Cadence was affected only in 3/20 (15%) patients and the time spent during stance phase was observed to be within normal limits for all participants. However, in the statistical analysis comparing the mean values for the two groups using the Mann Whitney U test (adjusted for multiple comparisons), only the gait velocity (mean 99.1 ± 26.3 vs. 124.1 ± 20.3; *p* = 0.001) and reduced step length (mean 57.2 ± 10.6 vs. 70.2 ± 10.3; *p* = 0.001) were significantly different in the DT group compared to healthy controls (Figure 1).

### Clinical features of DT participants and relationship with gait findings

Age of the DT participants was found to correlate significantly with their TUG time (r = .49; *p* = 0.01), 10 m gait speed (r = −.473; *p* = 0.015), score on the BBS (r = −.537; *p* = 0.015) and the gait velocity (r = - 0.479; *p* = 0.018) measured on the walkway system. However, gender, disease duration, head tremor severity, presence of axial tremors such as jaw tremor and voice tremor, MoCA score and botulinum doses did not impact the gait findings measured with clinical scales (GFQ, BBS, 10 m walk and TUG time) as well as the instrumented walkway system (velocity, cadence, step time, step length, stride width, percentage of time spent during swing and support phase, single support phase and double support phase and the gait variability index (*p* > 0.05).

### Comparisons of DT vs. ET and DT vs. OT

Demographics and gait findings of ET and OT groups are presented in Table 3. The ET group consisting of patients with bilateral arm tremors also had five patients with additional head tremors. In the OT group, 4 participants complained of bilateral arm tremors, and none had a head tremor. There were more females in the DT group compared to ET (17 vs. 6; *p* = 0.01) and the OT group (17 vs. 10; *p* = 0.04). There were no significant differences in age and MoCA scores. Disease duration was significantly longer for the OT group than the DT group (29.6 ± 8.3 vs. 17.6 ± 9.1; *p* = 0.04). In the gait and balance testing, time needed to complete the TUG testing was longer (13.6 ± 3.5 vs. 10.7 ± 2.3; *p* = 0.03) and scores recorded on the BBS were reduced (45 ± 4.7 vs. 50.1 ± 4.5; *p* = 0.02) in the OT group compared to the DT group. In the instrumented gait analysis, the cadence was reduced in ET (95.1 ± 11.2; *p* = 0.04) and OT (89.3 ± 9.8; *p* = 0.03) compared to DT (103.4 ± 10.3). The step length (51.4 ± 6.7 vs. 56.7 ± 7.8; *p* = 0.02) and GVI (89.1 ± 7.1 vs. 118.4 ± 8.7; *p* = 0.01) were reduced, and the time spent during the double support phase (35.9 ± 15.1 vs. 32.1 ± 15.2; *p* = 0.045) was increased in OT compared to DT (Figure 1).

## Discussion

Our study demonstrates that cervical dystonia patients with co-occurring head tremors display a number of spatiotemporal abnormalities related to gait. Although it has been previously suggested that gait impairments can be seen in cervical dystonia, the inclusion of head tremors as a clinical presentation has not been taken into account [18, 19]. The DT group in our study walked slower with shorter steps and with a broader base, and spent relatively greater amounts of time during the double limb support phase of the gait cycle. Many DT patients revealed that the gait variability was increased. Although the BBS scores for balance assessment were mainly within normal limits, nearly 40% of patients needed more time to complete the TUG test. Our study also found that among the tremor disorders, the most impressive number of abnormalities were present in the OT group compared to DT and ET. Patients with OT needed the highest amount of time on TUG, had relatively worsened BBS scores, walked slower with shorter steps, spent much more time during the double support phase, and had a higher gait variability. These findings support the shared link to the cerebellum as the source of tremor pathogenesis and gait dysfunction and emphasize the need for involving rehabilitation care when managing patients with tremor disorders in clinical settings.

Three-fourths of our DT cohort were females, which is not surprising as cervical dystonia affects females more frequently [20]. An increased preponderance of head tremors is also observed in females with cervical dystonia [21–23]. The findings of increased TUG seen in the DT cohort raise concerns that there is decreased control of mobility, transfers, and balance and they may be an increased risk of falls. Indeed, patients with cervical dystonia have been found to display deficits in balance, gait, and stepping reactions and they have expressed a higher fear of falling [12, 24]. In our study, many DT patients were observed to have an increase in step time, stride width, and time spent during double support to attempt increasing the stability during walking [25]. A lower walking speed in our cohort may have allowed the patients to maximize the sensory feedback from the lower limbs to aid in stability and balance. These natural adaptations have been noted to commonly occur in many other neurological populations such as multiple sclerosis [25, 26]. We also observed that as the age of DT patients increased, there was further lowering of gait speed and a concomitant increase in the time needed to complete the TUG task. We believe, a worsened age may have accelerated the progression of pathological changes in the tremor network leading to worsening of findings. Similar to our findings, a previous study in cervical dystonia found subclinical abnormalities such as increased gait variability and lower gait velocity [10]. However, it was not clear whether the patients in that study had a head tremor in addition to abnormal neck posturing.

Many potential hypotheses could be conjectured to explain the gait and balance findings observed in our DT cohort. A sustained, aberrant neck position appears to reduce the reliability of visual cues for postural control which, in turn, negatively impacts balance and balance related confidence [24]. As such, an abnormal head posture in cervical dystonia has also been found to impact vestibular functions [27] and proprioceptive capabilities [28, 29]. Another consideration is related to cervico-collic and tonic neck reflexes which may be affected and these factors could influence head, eye, and postural stability [30, 31]. In keeping with this hypothesis, a previous study found that patients with cervical dystonia have an increase in postural sway when standing [26]. In one study, a reduced range of motion for the cervical spine was found to correlate with balance and stepping reaction time in cervical dystonia [11]. A number of studies have also drawn attention to the orthopedic and spinal cord complications emerging from chronic mechanical stress of cervical dystonia related to constant twisting motion [32, 33]. Reduced control over voluntary neck movements is expected to render navigating complex environments challenging While the presence of head tremors and the resulting mechanical instability is undoubtedly important, previous research supports a pathogenic role of the cerebellum, particularly in the context of DT [34]. Many participants in our DT cohort walked with a slower speed, revealed an increased stride width, and spent more time in the double support phase of the gait cycle, findings similar to those seen in patients with cerebellar dysfunction [9, 35]. Thus, many factors in varying combinations can potentially explain the gait and balance findings in our DT cohort.

Interestingly, only 20% of our DT group reported clinical difficulties with gait and balance, indicating that the changes noted in our study were subclinical for most patients. Further, the severity of head tremors in the DT cohort was not found to predict gait and balance abnormalities. Our study cannot parse out whether the gait abnormalities are compensatory, or consequential. We also think that the relationship between cervical dystonia and gait is bidirectional, as sometimes, we observed a worsened dystonic posturing of the neck when the patients performed the gait task (Supplementary Video). Thus, it is possible that cervical dystonia leads to worsening of gait, and the performance of gait task exacerbates symptoms of cervical dystonia.

The cerebellum has been regarded as one of the key sources of pathogenic oscillations in other tremor disorders such as ET and OT [36–39]. In the context of ET, presence of head tremors has been found to predict gait dysfunction and balance abnormalities [40]. In a large study of ET patients, axial tremors, including the presence of head and jaw tremors, were associated with significant tandem gait disturbances [41]. Previous studies have reported that OT patients have abnormalities in postural balance assessments [42, 43], and spatial and temporal characterizations of gait [44]. With disease advancement, patients with OT have been observed to walk with shorter steps and a wider base, and spend more time during the double support phase. These patterns of gait abnormalities are similar to those seen in patients with cerebellar disorders [45]. Our study also found notable abnormalities in gait variability in OT patients. Gait variability, defined as the fluctuation in spatiotemporal characteristics between steps, is suggested to be a sensitive indicator of mobility deficits with pathological processes [46]. Some investigators report gait variability of spatial parameters, for example, the variability of the stride width to be a more important indicator of locomotion control than gait variability of temporal parameters. In our study, OT patients had greater gait and balance abnormalities compared to DT patients, which could be due to the fact that these patients are in general older in age and had longer disease duration.

We acknowledge that our study has limitations. While the DT group specifically had a head tremor, our control groups comprising OT and ET did not necessarily share the same phenotype (head tremor present only in a subgroup of ET). Further, the sample size for DT in our study was relatively small; we did not characterize and examine whether the severity of dystonia or electrophysiology of head tremor could impact the gait findings, we did not address the issues of postural sway and near falls, and we have not examined the gait under cognitive loading. We recognize that the intake of GABAergic medications by the patients in our study could have influenced our gait findings as these medications affect cerebellar functions. Although we did not specifically use a statistical model to adjust for medication doses, we collected all data when the patients were off medications to minimize the impact on data interpretation.

Nevertheless, our study has unique strengths as it is the first to focus on the presence of head tremors and their potential impact on gait and balance assessments in cervical dystonia. It compares these findings with other tremor disorders that share cerebellar pathology. Future studies with larger cohorts of dystonia patients with and without tremors as well as plans for longitudinal follow-up, are needed to confirm our findings. Future studies should involve EMG recordings from the neck and leg muscles in conjunction with the instrumented walkway system to understand the relationship between dystonia and gait. It would be interesting to investigate whether gait and balance abnormalities are unique to specific dystonia subtypes, as the pathogenic mechanism is quite heterogeneous. Studies with such designs and cohorts will advance our understanding of the cerebellum and its control over dystonia, tremor, and gait. Importantly, our study findings inform clinicians that rehabilitation strategies should be given due consideration for managing tremor disorders in the outpatient settings.

## Supplementary Material

Supplementary Table

Patient 1 Video SUPPLEMENTARY VIDEO S1
A case of DT walking on the Zeno gait mat with significant torticollis to the right. The patient exhibits head tremors when walking. The patient voluntarily corrects his head position to the center as he pauses and takes a turn (segment 16 s to 19 s). His head remains stable for a few seconds, however, involuntarily pulls to the right as he continues to walk (segment 21 s to end).

Patient 2 Video SUPPLEMENTARY VIDEO S2
A case of DT with significant anterocollis and torticollis to the right. The video is recorded while performing the TUG task. Patient tends to take support from the wall as she walks towards the chair with slight unsteadiness (6 s to 7 s). She uses a sensory trick to steady her head (29 s to 32 s). However, as she continues to walk without using the trick (33 s onwards) her head posture worsens.

## Figures and Tables

**TABLE 1:** Clinical characteristics of the DT cohort.

Pt	Age in yrs	Sex	Disease duration in yrs	Body region affected by dystonia	Body region affected by tremor	Head tremor severity	Oral medications	BoNT	BoNT dose in units	Gait & freezing questionnaire	TUG time (s)	10 m walk (m/s)	MoCA	Berg balance test

1	75	F	25	neck	head, arms	3	clonazepam, gabapentin	y	160	12	16.3	0.6	25	47
2	79	M	15	neck, jaw	head, jaw, arms	2	metoprolol, alprazolam	n	0	0	10.3	1.2	24	53
3	73	F	4	neck	head	1	THP	n	0	14	12.1	0.8	22	52
							MT							
4	60	M	5	head, neck	head	1	eszopiclone, paroxetine, clonazepam	Y	300	6	8.8	1.3	24	55
5	82	F	22	neck, eyes	head	2	clonazepam	y	200	14	12.2	0.9	26	47
6	80	F	15	neck	head	1	primidone, propranolol	y	200	6	8.4	1.3	27	49
7	50	F	2	neck	head	1	none	y	200	0	8.3	1.2	27	56
8	85	F	20	neck, larynx	head, voice, arms	3	primidone, propranolol	y	225	4	12.5	0.8	24	46
9	65	F	14	neck	head, arms	2	alprazolam, metoprolol	y	260	1	9.15	1.2	26	49
10	67	F	12	neck	head, arms	2	propranolol	y	200	8	12.5	0.9	27	47
11	63	M	4	neck	head	1	clonazepam	y	300	7	14.3	0.9	25	44
12	71	F	12	neck, jaw, arms	head, arms	1	zolpidem, metoprolol, CBZ	y	400	14	13.1	0.8	29	45
13	69	F	5	neck	head	1	none	y	250	0	11.7	1.2	23	39
14	55	M	25	neck	head	2	gabapentin	n	0	3	8.4	1.3	24	54
15	61	M	50	neck, larynx	head, voice	3	clonazepam, zolpidem, propranolol	y	400	7	9.9	1.1	25	55
16	64	F	16	neck	head	2	primidone, propranolol, clonazepam	y	380	7	15.7	0.7	25	45
17	66	F	3	neck	head	1	primidone, propranolol, clonazepam, benztropine	y	200	2	10.7	1.1	22	49
18	66	F	50	neck	head	2	clonazepam, tizanidine	y	255	5	8.6	1.1	20	55
19	64	F	24	neck	head, arms	2	baclofen, clonazepam	y	400	0	8.5	1.2	27	53
20	41	F	20	neck	head	2	CBZ	y	300	0	6.6	1.4	28	56

THP, trihexyphenidyl.

CBZ, cyclobenzaprine.

MT, methocarbamol.

**TABLE 2:** Gait data for DT recorded with instrumented walkway system.

Pt	Age in yrs	Velocity cm/sec	Cadence steps/min	Step time (sec.)	Step length (cm.)	Stride width (cm.)	Swing %	Stance %	Single support %	Total D. support %	GVI

1	75	**54.9**	**85.3**	**0.72**	**38.6**	8.8	**32.2**	68.7	**31.6**	**36.6**	**138.9**
2	77	113.7	103.2	0.53	67.2	**18.2**	**32.9**	63.5	36.6	29.3	**130.8**
3	71	**69.6**	103.8	**0.67**	**42.3**	10.9	**30.9**	68.8	**31.4**	**37.9**	**130.3**
4	60	**95.8**	98.0	**0.69**	56.7	12.1	34.9	65.3	35.0	30.8	110.2
5	80	**126.5**	117.0	0.53	63.8	10.5	37.8	63.6	36.6	25.8	117.0
6	50	**115.9**	110.5	0.54	63.9	12.0	34.7	64.8	35.3	30.1	110.0
7	85	**78.7**	103.7	0.61	48.5	13.8	32.5	64.1	36.5	30.9	**131.1**
8	65	117.0	102.2	0.60	69.7	11.4	34.5	66.5	33.2	**32.4**	113.2
9	67	**87.9**	100.9	**0.64**	56.6	**17.0**	**29.5**	69.4	**30.8**	**38.9**	**136.5**
10	63	**78.7**	**87.0**	**0.72**	54.1	14.4	34.0	67.7	**32.5**	**33.8**	119.9
11	71	**72.9**	101.5	**0.68**	**42.8**	13.4	33.2	68.2	**31.7**	**34.7**	**132.2**
12	69	**93.8**	106.5	**0.66**	52.5	**15.8**	**32.3**	65.7	33.9	33.6	**132.7**
13	55	**91.1**	100.1	0.62	52.9	10.2	**32.0**	66.9	33.3	32.9	118.5
14	61	101.6	96.8	0.61	60.9	**18.6**	34.6	65.4	34.6	30.5	111.0
15	64	**78.7**	103.0	**0.66**	**46.3**	10.7	33.4	69.7	**30.5**	**36.3**	**135.6**
16	66	**77.0**	91.3	**0.73**	49.4	9.1	31.6	67.7	**32.2**	**36.1**	107.3
17	66	121.6	114.9	0.52	64.9	10.2	34.3	65.7	34.4	31.0	100.0
18	64	138.2	126.3	0.54	66.7	8.6	35.6	63.6	36.4	27.8	97.6
19	41	137.0	113.4	0.55	73.8	12.8	37.8	62.3	37.8	24.7	100.0
20	65	131.8	107.4	0.61	74.3	9.1	36.7	62.8	37.2	25.8	97.6

Bold values are abnormal values for individuals when comparing to age and sex matched healthy control values.

**TABLE 3:** Comparisons of gait data in DT with ET and OT cohorts.

	Dystonic tremor (DT)	Essential tremor (ET)	Orthostatic tremor (OT)	DT vs. ET (*p*-value)	DT vs. OT (*p*-value)

Number of participants	20	15	15		
Age in years (mean ± SD)	66.5 ± 8.9	68.8 ± 7.8	70 ± 6.5	*p* = 0.71	*p* = 0.23
Sex (Male: Female)	3:17	9:6	5:10	**p = 0.01**	**p = 0.02**
Disease duration in years (mean ± SD)	17.6 ± 9.1	22.5 ± 8.9	29.6 ± 8.3	*p* = 0.06	**p = 0.04**
MOCA score	24.4	23.1	28.5	*p* = 0.56	**p = 0.04**
Gait and freezing questionnaire total score (mean ± SD)	4.2 ± 1.4	4.8 ± 1.5	4.5 ± 2.1	**p = 0.057**	*p* = 0.63
TUG walking time in seconds (mean ± SD)	10.7 ± 2.3	12.8 ± 3.1	13.6 ± 3.5	**p = 0.05**	**p = 0.03**
10 m walk (speed) in m/seconds (mean ± SD)	0.9 ± 0.2	0.9 ± 0.2	0.7 ± 0.3	*p* = 0.82	*p* = 0.13
Berg Balance total score (mean ± SD)	50.1 ± 4.5	48.9 ± 4.6	45 ± 4.7	*p* = 0.47	**p = 0.02**
Gait velocity in cm/seconds (mean ± SD)	99.1 ± 9.1	97.6 ± 8.4	96.8 ± 8.9	*p* = 0.78	*p* = 0.67
Cadence in steps/minute (mean ± SD)	103.4 ± 10.3	95.1 ± 11.2	89.3 ± 9.8	**p = 0.04**	**p = 0.03**
Step time in seconds (mean ± SD)	0.68 ± 0.2	0.64 ± 0.2	0.81 ± 0.5	*p* = 0.71	**p = 0.06**
Step length in cm (mean ± SD)	56.7 ± 7.8	59.0 ± 9.1	51.4 ± 6.7	**p = 0.13**	**p = 0.02**
Stride width in cm (mean ± SD)	12.4 ± 4.5	14.8 ± 5.6	14.9 ± 6.9	*p* = 0.28	*p* = 0.16
Swing % (mean ± SD)	33.8 ± 12.1	32.8 ± 11.4	32.1 ± 13.4	*p* = 0.68	*p* = 0.79
Stance % (mean ± SD)	66.1 ± 12.3	67.2 ± 13.4	67.9 ± 12.5	*p* = 0.77	*p* = 0.62
Single support % (mean ± SD)	33.9 ± 14.1	32.9 ± 13.1	32.1 ± 14.6	*p* = 0.71	*p* = 0.17
Double support % (mean ± SD)	32.1 ± 15.2	34.3 ± 13.4	35.9 ± 15.1	**p = 0.12**	**p = 0.045**
Gait variability index (GVI) (mean ± SD)	118.4 ± 8.7	95.1 ± 8.6	89.1 ± 7.1	**p = 0.04**	**p = 0.01**

Bold values indicate significant *p* values.

## Data Availability

The raw data supporting the conclusion of this article will be made available by the authors, without undue reservation.
