# Supplementary material for: Gait and balance in cervical dystonia and dystonic head tremor
Source: Dystonia. Author manuscript; Available in PMC 2025 Jul 24. (PMC12288549; doi:10.3389/dyst.2023.11231)
Supplement: Supplementary Table [file NIHMS2068339-supplement-Supplementary_Table.docx]

Table: Gait data collected in neurologically healthy individuals (n = 46) with instrumented walkway system

|  | velocity (cm/sec) | | cadence (steps/min.) | | step time (sec) | | step length (cm) | | stride width (cm) | | swing (%) | | stance (%) | | single support (%) | | double support (%) | | GVI |  |
| --- | --- | --- | --- | --- | --- | --- | --- | --- | --- | --- | --- | --- | --- | --- | --- | --- | --- | --- | --- | --- |
| Age in years | min | max | min | max | min | max | min | max | min | max | min | max | min | max | min | max | min | max | min | max |
| 45 - 50 | 125.1 | 159.9 | 100.8 | 113.9 | 0.53 | 0.62 | 70.6 | 84.1 | 6.9 | 13.1 | 35.1 | 39.7 | 58.3 | 66.9 | 35.1 | 39.7 | 21.9 | 28.7 | 95 | 118 |
| 50 - 55 | 120.1 | 159.9 | 99.3 | 112.7 | 0.53 | 0.62 | 70.3 | 85.2 | 6.4 | 12.8 | 34.9 | 39.8 | 57.9 | 67.4 | 34.9 | 39.8 | 21.5 | 29.4 | 94 | 119 |
| 55 - 60 | 115.2 | 149.9 | 98.3 | 116.6 | 0.52 | 0.61 | 65.4 | 80.4 | 6.4 | 13.7 | 34.2 | 40.1 | 57.3 | 68.7 | 34.2 | 40.1 | 22.1 | 30.1 | 96 | 122 |
| 60 - 65 | 101.4 | 133.4 | 94.1 | 110.2 | 0.54 | 0.64 | 61.1 | 77.1 | 6.7 | 12.7 | 35.1 | 38.1 | 61.1 | 65.3 | 35.3 | 38.9 | 23.3 | 29.3 | 86 | 124 |
| 65 - 70 | 107.3 | 137.3 | 96.3 | 116.1 | 0.51 | 0.61 | 61.3 | 75.2 | 6.7 | 14.1 | 35.2 | 38.2 | 61.5 | 66.5 | 35.3 | 38.7 | 24.2 | 28.8 | 91 | 124 |
| 70 - 75 | 95.1 | 129.2 | 95.2 | 111.6 | 0.55 | 0.67 | 57.3 | 73.3 | 7.2 | 15.2 | 33.8 | 39.4 | 61.1 | 66.5 | 34.3 | 38.7 | 22.7 | 32.1 | 90 | 126 |
| 75 - 80 | 79.4 | 123.1 | 91.1 | 113.3 | 0.51 | 0.64 | 49.4 | 69.2 | 5.1 | 14.7 | 32.4 | 37.8 | 62.2 | 67.6 | 33.1 | 37.3 | 26.8 | 33.8 | 89 | 121 |
| 80 - 85 | 79.1 | 118.5 | 89.4 | 103.3 | 0.51 | 0.62 | 49.1 | 65.2 | 5.0 | 14.9 | 32.4 | 37.8 | 60.2 | 66.8 | 30.1 | 38.9 | 24.8 | 36.9 | 89 | 121 |

GVI: gait variability index
